# Retraction: Oxidative Stress and Mitochondrial Functions in the Intestinal Caco-2/15 Cell Line

**DOI:** 10.1371/journal.pone.0267058

**Published:** 2022-04-14

**Authors:** The PLOS ONE Editors

Following the publication of this article [1], concerns were raised regarding results presented in Figs 6, 8, and 9. Specifically,

- The following bands appear similar:
  ○ Fig 6 AIF panel, lanes 1 and 3, and Fig 8 right OGG1 panel, lane 2.
  ○ Fig 6 AIF panel, lane 2, and Fig 8 right OGG1 panel, lanes 1 and 3.
  ○ Fig 6 left β-actin panel, lanes 1, 2, 3 and 4.
  ○ Fig 9A far left GAPDH panel, all lanes, and Fig 9A MtTFB1 panel lane 2.
- When levels are adjusted to visualize background, there appear to be vertical irregularities suggestive of splice lines in:
  ○ Fig 6 Cytochrome-C panel between all lanes.
  ○ Fig 8 right OGG1 panel, between lanes 1–2, and lanes 3–4.
  ○ Fig 8 left OGG1 panel, between lanes 1–2, and lanes 3–4.
  ○ Fig 9A middle left GAPDH panel, between lanes 3–4.
  ○ Fig 9A middle right GAPDH panel, between all lanes.
  ○ Fig 9A POLRMT panel, between lanes 3–4.
  ○ Fig 9A far right GAPDH panel, between lanes 1–2 and lanes 2–3.
  ○ Fig 9B MtTFA panel, between lanes 2–3.
  ○ Fig 9B far left β-actin panel between lanes 3–4.
  ○ Fig 9B MtTFB1 panel and corresponding β-actin panel, between lanes 2–3.
  ○ Fig 9B far right β-actin panel between lanes 2–3 and lanes 3–4.

The corresponding author stated that some images were spliced during figure preparation to remove extra lanes and/or replicates. They commented that data files were provided to PLOS in 2014–2015, but the underlying data for the figures of concern are no longer available in the laboratory records. PLOS is unable to access the journal’s 2014 correspondence records for this case. We sincerely regret that this case was not resolved much sooner after the prior correspondence.

The *PLOS ONE* Editors retract this article. Although we have been unable to review the primary data, the nature and extent of image concerns call into question the integrity of data reporting in the published figures and the reliability of the article’s results and conclusions.

GM, FB, and FPG agreed with the retraction and apologise for the issues with the published article. JFB, ED, and EL did not agree with the retraction. RT, DM, and DA either did not respond directly or could not be reached. ES is deceased.
